# Optimization of Extrusion-Based 3D Printing Process Using Neural Networks for Sustainable Development

**DOI:** 10.3390/ma14112737

**Published:** 2021-05-22

**Authors:** Izabela Rojek, Dariusz Mikołajewski, Marek Macko, Zbigniew Szczepański, Ewa Dostatni

**Affiliations:** 1 Institute of Computer Science, Kazimierz Wielki University, 85-064 Bydgoszcz, Poland; izarojek@ukw.edu.pl (I.R.); dmikolaj@ukw.edu.pl (D.M.); 2 Department of Mechatronic Systems, Faculty of Mechatronics, Kazimierz Wielki University, 85-064 Bydgoszcz, Poland; mackomar@ukw.edu.pl (M.M.); zszczep@ukw.edu.pl (Z.S.); 3 Institute of Materials Technology, Faculty of Mechanical Engineering, Poznan University of Technology, pl. M. Skłodowskiej-Curie 5, 60-965 Poznań, Poland

**Keywords:** optimization, 3D printing, sustainable development, neural networks

## Abstract

Technological and material issues in 3D printing technologies should take into account sustainable development, use of materials, energy, emitted particles, and waste. The aim of this paper is to investigate whether the sustainability of 3D printing processes can be supported by computational intelligence (CI) and artificial intelligence (AI) based solutions. We present a new AI-based software to evaluate the amount of pollution generated by 3D printing systems. We input the values: printing technology, material, print weight, etc., and the expected results (risk assessment) and determine if and what precautions should be taken. The study uses a self-learning program that will improve as more data are entered. This program does not replace but complements previously used 3D printing metrics and software.

## 1. Introduction

The ability to handle advanced technologies requires increased attention to education, shaping the technological awareness of the public, and a high level of organizational capacity to manage such emerging complexity. Sustainability within three-dimensional (3D) printing technology (also known as additive manufacturing) should consider the use of materials, energy, emitted particles, and waste. The lower prices of 3D printers (even under $100) and materials make these devices and related technologies accessible and useful to many people. Various authors have mentioned that the possibilities of digital fabrication [1,2] are enhanced by a widespread use of technology. It can be predicted that many people will use this technology, generating a lot of waste in the near future. Therefore, the novel problems of 3D printer energy consumption, improperly disposed or recycled materials, and harmful emission rates require urgent diagnosis, monitoring, and effective solutions. Environmental pollution of plastic waste is a serious problem due to its non-degradability [3,4,5].

The overall problem is much deeper: with such novel emerging technologies, we cannot assume that the current level of use of particular materials is the critical variable determining the development of a given state of affairs. On the contrary, we must be aware that the fundamental issue will be relatively new values based on the evolving competencies of people and the ability of companies to design unique, cutting-edge technology products and services. Intellectual property (IP) rights and know-how for such innovative solutions offering an advantage in the market will be difficult to achieve. Instead of developing the technology ourselves, we can buy it, but that market is usually created by a narrow community that gathers both the knowledge of the technology and the opportunities for its purposeful use. There is a strong link between inventions and their application and commercialization. The market synergy between competencies and components requires a complex solution supported by computational intelligence (CI)-based analytics to manage such complexity. Other factors that increase the aforementioned system complexity include: the need for a deeper understanding of the productive collaboration and hidden dependencies of interdisciplinary teams, the rationale for overcoming communication barriers that limit transparency, simultaneous production of complementary solutions based on a (seemingly) unified architecture and a decentralized collaboration system, and management methods based on task mapping and easy data sharing [6].

New approaches, including 3D technologies, the Internet of Things (IoT), Industry 4.0, etc., are bringing continuous development and change, shaping our current approach to technology as a simple factor in changing the way we live our daily lives [7,8]. Products are shaped by communities or individuals according to their needs to improve their quality of life, and once they achieve that—they immediately start looking for something more advanced, helpful, interesting, etc.

The amount of data in itself does not matter much; only consciously interpreted and processed data become information and, only in some cases, new knowledge. Using the right tools to analyze 3D printing reverse engineering data allows you to gather information that is important to scientific progress or the operation of your business: to gain valuable insights, support important decisions, and develop new or significantly improved products or services. In some cases (technical inspection) they help to avoid losses. The mentioned data analysis is thus an answer to the research problem posed. It can be explicitly formulated, as well as stated more generally, e.g., in the form of a hypothesis [9].

Organizations that efficiently collect and use data are doing better and better on the market, and in some industries it is essential for market success. Establishing quantitative relationships between phenomena allows you to draw more accurate conclusions and, based on them, make decisions with fewer errors in the areas of quality control, monitoring error rates, improving operational efficiency, reducing costs, analyzing product lines, increasing revenue, evaluating sales, analyzing and reporting compliance, and evaluating projects to identify new opportunities or hidden problems [10].

“Manual” data analysis required extensive knowledge of statistics and programming. Automated or semi-automated data analysis based on artificial intelligence is much easier, faster, cheaper, and more efficient. In the case of 3D printing, we are dealing with multi-criteria optimization, which involves finding an optimal solution that is acceptable from the point of view of each of the selected criteria. In principle, the optimization problem can be formulated in a rigorous manner as long as we are able to define the objective function, also known as the quality criterion, a model of the phenomenon with distinctive decision variables, and constraints. Here, there are, among others:

- optimization of business processes—involves designing improvements in the functioning and management of the enterprise by reorganizing the processes taking place inside the company,
- construction optimization—deals with issues related to the selection of the parameters of the physical features and shape,
- optimization of logistics processes—concerns the maximum use of the company’s logistics resources, with particular emphasis on time, costs, efficiency, and flexibility of processes,
- production optimization—is based on reducing production costs, mainly due to the reduction of its duration, and is also an opportunity to gain better control over the production process, reduce the number of defective products, and use the full production potential more effectively,
- cost optimization—helps the company to save as much money and resources as possible so that the company does not run at a loss and benefits as much as possible,
- software optimization—is an activity consisting of the analysis and improvement of a computer program by increasing the efficiency of operation and reducing the use of computer power [11,12].

Three-dimensional printers are becoming more and more economical as the technology evolves. Therefore, we introduce a new AI-based software to assess the amount of pollution generated by 3D printing systems. We enter input values: printing technology, material, print weight, etc., and predict the results (risk assessment) and determine if and what precautions should be taken.

### 1.1. Optimization of 3D Printing

The idea of optimizing 3D printing is central to the development of this group of technologies by relying on new printing technologies, new mechanical properties of materials, and automation of their use in 3D printing (including multi-material printing) [13]. Automation allows for more accurate consideration of input parameters and requirements for printed object properties and applications [14]. It is important to determine the relationship that exists between 3D printing process parameters, performance and durability, the quality of the printed object, and its structural properties [15].

Optimization of 3D printing parameters allows for cost and time reduction of additive manufacturing and associated processes (e.g., reverse engineering) [16]. Three-dimensional printing optimization typically reduces the cost of short production runs, tooling, and, most importantly, prototypes [17]. To achieve this, many parameters must be taken into account: the volume and dimensions of the object(s), the amount and type of material used, the working time during the entire process, etc. [18]. This can make 3D printing much cheaper compared to parallel technologies: milling, mold-making, and mass production [19]. Generally, the cost of the printed part is affected by the following factors:

- costs of preparing a 3D print—including preparation of the printer for work, measurements required for providing the appropriate 3D printing environment, implementation of printing material [20], choosing optimal temperature settings (for a given material, sample size, and other requirements such as direction of working for the best durability, etc.) [21], speed [22], and additional process parameters [23],
- model volume [24]—including size of the object(s) [25], amount of space inside the device to be printed [26], time required for a complete printout [27],
- time cost of 3D printing [28]—including the number of working hours of the 3D printer using a given technology, production capacity, printer depreciation cost, and all service activities or expenses related to the proper operation of the 3D printer [29], and sometimes availability and occupancy of the data of the 3D printers (e.g., quicker printing may cost extra) [30],
- costs of finishing the 3D print—including grinding, impregnation (e.g., with epoxy resin), joining with metal components, painting, gluing [31]—additional processing of the model may be possible at the customer’s special request [30],
- cost of electricity [31],
- cost of the operator and post-processing technician [32].

The average 3D printing time depends primarily on the technology used and the material chosen. Due to the above issues, industrial 3D printers can sometimes be replaced by cheaper printers with a lower initial purchase cost.

The literature on 3D printing process optimization is abundant, especially in the last 10 years. Many important advances have been made in the aforementioned studies, but few of them address the problem of CI (Computational Intelligence)-based energy and environmental optimization.

The default settings of printing process parameters in some cases do not guarantee the quality (as described by the dimensions of error, strength, etc.) of the printed objects. In a study by Pawar et al. [33], three parameters of the FDM (Fused Deposition Modelling) printing process (layer thickness, layer speed, and fill density) were optimized using the Taguchi L9 Orthogonal Array method. More layers will result in a high temperature gradient toward the bottom of the part, which will increase diffusion between adjacent rasters and improve strength [33]. A low layer height of 0.14 mm allows for the shortest printing time, assuming print quality assurance [34]. New possibilities for material optimization are opened by composite materials with specific mechanical properties. Thus, several materials can be used in one manufacturing process. Researchers aim to create a database for the analysis and implementation of material properties in order to develop a proper common approach for the selection of material(s), and also in terms of the geometric perfection of printed objects [35]. Camposeco-Negrete [36] proposed a unified approach to optimize five FDM-related responses: power consumption of the 3D printer, processing time, dimensional accuracy of the part, amount of material used to print the part, and mechanical strength of the samples.

### 1.2. Energy Consumption due to 3D Printing

The gradual greening of the 3D printing industry continues to grow, driven by the optimization of the energy efficiency of the machines used to print various objects and the worldwide trend to make 3D printers environmentally friendly devices. The main advantages expected from 3D printing include reduced environmental impact, including lower material and energy consumption compared to traditional manufacturing methods. This is mainly due to better adaptation of the aforementioned technology to the single end user of the items produced in this way, without the need to produce components in other sizes or spare parts for them. It is also assumed that there will be less waste, and some or even all of it will be recycled. Simon et al. experimentally investigated energy consumption and air emissions during 3D printing (FDM). In FDM, most of the electricity is used to heat the print bed and maintain its temperature. It is possible to reduce particulate emissions during FDM printing by changing procedures and process parameters [37].

The average power consumption of a traditional 3D printer usually does not exceed a few hundred watts. In general, values for 3D printers printing with PLA (polylactic acid), PLA + (160–222 °C) are lower than those for 3D printers printing with ABS (Acrylonitrile butadiene styrene) due to the lower melting point of the former two materials. Other melting points are as follows: polyhydroxyalkanoates (PHA): 190–210 °C; polyvinyl alcohol (PVA): 160 °C; polyethylene terephthalate (PET): 190–210 °C; and high impact polystyrene (HIPS): 210–230 °C. Higher power consumption can be expected in machines with a larger heated bed or more extruders. Moreover, 3D printers with a closed working chamber can be considered more energy-efficient. The upper limit of a printer’s power consumption is set by the power of the power supply itself, but the printer does not run at full power all the time. Various components of the 3D printer consume different amounts of electricity at different times, depending on the work stage, i.e., from the most power-consuming parts of the 3D printer to the least:

- the heated bed creates the highest power consumption: up to more than approximately 60% of the limit of the power supply,
- less electricity is needed to melt the filament in the head,
- even less for the stepper motors,
- less for fans,
- and at the end are the electronic devices.

Power consumption during the work cycle is usually as follows (mean values with SD compared to the power of the power supply with SD):

- 3 ± 0.5%—when the only printer is powered,
- 65 ± 10.1%— with only the table heating on,
- 23 ± 2.2%—with hotend heating on (and the extruder cooling fan on),
- 5 ± 0.8%—with its own fans for cooling the print, set to the maximum speed,
- 6.5 ± 1.2%—during operation of all of the stepper motors,
- 85 ± 13.2%—while heating the table and nozzle, and all of the fans are on.

The same relative to the highest consumption (mean ± SD):

- 3.5 ± 0.6%—when only the printer is powered,
- 76.5 ± 11.9%— with only the table heating on,
- 27 ± 2.6%—with hotend heating on (and the extruder cooling fan on),
- 5.9 ± 0.9%—with its own fans for cooling the print, set to the maximum speed,
- 7.6 ± 1.4%—during operation of all of the stepper motors,
- 100 ± 15.5%—while heating the table and nozzle, and all of the fans are on.

The average cost of electricity for 3D printing is a few zlotys per hour (about EUR 1= 4.47 PLN-Polish zloty)—thus 3D printing seems to be one of the more cost-effective methods of manufacturing objects. You have to take into account the lack of production facilities beyond a computer with a design and a 3D printer (sometimes also a 3D scanner). Energy-efficient printers are still being developed—there are at least a few solutions that make this possible: reduction of energy losses, reduction of the extruder weight, which allows for faster and more precise printing, better distribution of heat from the heating element to the printer compartment, and use of a curtain to divide the working chamber—the heated air is used only in the area where the model is being printed—or even the option to change the size/volume of the working chamber [37,38]. The last option is considered the most efficient and optimal, for printing both larger and smaller objects, for prototyping, short and very short runs, and for mass production.

To facilitate the energy consumption decision-making process, we tried to generalize the precautions using our artificial neural network (ANN)-based reasoning system.

### 1.3. Air Pollution due to 3D Printing

Being in and breathing in rooms where 3D printers work can have harmful effects on a person’s respiratory system. During 3D printing, plastic is melted and then layered to form the desired shape of objects. The material is heated, which releases volatile compounds into the surrounding air. There is no doubt that both short- and long-term exposure to the particles released during the melting of the filament(s) can have negative health effects similar to those from exposure to urban air pollution. The toxic effects of the various filaments used in 3D printing technologies can seriously affect cell cultures of the human respiratory system and immune system cells. Both of the most common materials (ABS and PLA/PLA+) negatively affect cell viability, but PLA shows even more toxicity. The higher temperature needed to melt the filament implies stronger emissions of these compounds. PLA molecules are more toxic than ABS molecules, but the emission of ABS by printers is much higher.

It is clear that 3D printer rooms should be well and frequently ventilated, no one can stand close to the 3D printer while the 3D printers are running, and 3D printers should additionally have special safety chambers. In order to facilitate decision-making processes in the area of 3D printing-related air pollution, we have attempted to generalize the precautions using our artificial neural network (ANN)-based reasoning system.

Thermoplastics have been recycled since the 1970s, so there is already considerable knowledge and experience with the recycling process. Recycling by converting waste into new filaments is considered an effective recycling method, but the homogeneity of the source and similar resin properties are important. On the other hand, this recycling also means further degradation of the properties of the resulting filament, hence its full utilization is still a challenge. Despite the right attitude of manufacturers and consumers, the management, recycling, and disposal of materials in the 3D printing sector still need support and regulation, also in the area of state influence.

3D printing saves more material than traditional manufacturing processes. Other biodegradable plastics used for filaments (e.g., for Fused Deposition Modelling—FDM purposes) include PLA, and PLA+, PHA (polyhydroxyalkanoates, PVA (polyvinyl alcohol), PET (polyethylene terephthalate), HIPS (high impact polystyrene), and biocomposites (e.g., a biodegradable polymer matrix and about 40% by volume of bio-based fillers). It should be taken into account that the mechanical properties of biodegradable 3D-printed plastics or composites are not as good as pure matrix materials [39]. Thus, it can be concluded for the moment that sometimes the transformation of a material implies its degradation, and it should be used very carefully. From another point of view, this may be an opportunity for environmentally friendly applications of 3D printing with short life cycle. A novel framework for sustainability assessment and improvement of 3D printing processes by integrating computer-aided design (CAD) and life cycle assessment (LCA) was proposed by Liu et al. [40]. It seems that further research efforts should be focused on improving the feasibility of 3D printing using novel compostable or bio-based fibers.

Users may know little about the effects or impacts of pollutants (organic compounds and ultrafine particles) generated by equipment [41]. Moreover, the physical and chemical properties of the emitted dust remain unclear. Ultrafine particles and other hazardous materials are emitted during 3D printing, but the effect of temperature on these particles has not been systematically studied [41]. It is recommended to reduce particle emissions from 3D printing, print at the lowest possible temperature, and use low emission materials.

Measurement of the particle concentration with direct reading devices in the chamber at various temperatures (185–290 °C in steps of 15 °C) using four filament materials during 3D printing by FDM, taking into account the operating conditions recommended by the manufacturer, showed that:

- temperature was the key factor influencing the amount of emission by filament type,
- emission increased gradually with increasing temperature for all types of filament,
- emission value at the lowest operating temperature was 107–109 particles/minute,
- emission value at the highest temperature was 100–10,000 times greater [42].

ABS is much more toxic than PLA:

- emission of volatile organic compounds (VOCs) fluctuated within 0.50 µmol/h,
- styrene was responsible for over 30% of the total VOC emission from ABS,
- methyl methacrylate was responsible for over 44% of the total VOC emission from PLA [41].

Therefore, low emission materials are strongly recommended. However, recommendations for reducing particle emissions include not only using lower temperatures and using low-emitting materials, but also implementing control measures, using an enclosure/chamber around the printer, and using HEPA (high-efficiency particulate air) filters during 3D printing.

More stringent adherence to the manufacturer’s recommendations can result in a reduction in airborne particle counts; the nanoparticle emission factor is at least one order of magnitude higher for all fibers tested at a higher constant extruder temperature than at the lower temperature recommended by the manufacturer [43]. Long-term use of the printer also led to higher emission factors (factor 2 with PLA and factor 4 with ABS (Acrylonitrile butadiene styrene), measured after seven months of sporadic use) [44]. Furthermore, a single 3D print—even a long one (165 min) in a large, well-ventilated room—did not result in a significant increase in the concentration of harmful particles in the air, whereas such elevated concentrations of harmful particles were detectable indoors up to 20 h after printing in a small, unventilated room [45]. Even a 40-min 3D print can produce a harmful dose [45]. Aerosol emissions from nanoclusters (NCA) can account for 9–48% of total emissions, so up to half of particulate emissions may have been previously overlooked [46]. Diffusivity and extrusion rate are considered the most important variables in predicting environmental concentrations in the near field [47]. The aforementioned computational model would be useful for estimating worker exposure and for determining whether respiratory protection is necessary. The particles started to evaporate intensively at 150 °C, but only 25% of the particle number remained at 300 °C [48].

The growing concern about noise has accelerated the development of sound-absorbing devices [49]. VAT polymerization printers (SLA—stereolithography, DLP—digital light processing technologies) emitted nanoparticles containing potentially carcinogenic, allergenic, and reactive metals and carbonyl vapors. The observed differences in emissions between printers/technologies suggest that the technology used is an important factor in reducing exposure to harmful particles in the air [50]. Inhalation of fumes and organic particles containing metals is possible even when using 3D printing toys intended for children. Such toys should not be used in rooms with poor ventilation and/or placed near a child’s breathing zone [51]. In vitro cell studies and in vivo exposure in mice have shown toxic reactions induced by both PLA- and ABS-emitting particles (higher reaction levels) [52]. Emission rates can also be affected by printer failure, the type of filament used, and to a lesser extent the color of the filament [53]. Emissions released under non-industrial conditions can be potentially harmful. They can be mitigated by the use of a 3D printer shield, ventilation, and appropriate choice of filament composition and color. The simple use of a shield on a 3D printer reduces emissions by a factor of two [54]. There is no doubt that the concentration of particles reaches the highest values during heating and printing of the solid layer [55], for both ultrafine particles (UFP, <100 nm) and volatile organic compounds (VOCs) [56]. Average aerosol emissions range from 108 to 1011 particles per minute and vary during printing [57]. It is necessary to identify an objective marker that can accurately indicate the frequency, duration, and magnitude of exposure, such as measurement by optical particle counter (OPC) and condensation particle counter (CPC) [58]. However, even a high-efficiency particulate air filter installed in a 3D printer can be very useful [59], also for a laser printer [60] and to counter noise pollution [61]. Moreover, air pollution can be observed even after printing is completed. Training and developing proper habits and controls can be helpful.

The aim of this paper is to investigate whether the sustainability of 3D printing processes can be supported by computational intelligence (CI)- and artificial intelligence (AI)-based solutions.

## 2. Materials and Methods

The further development of 3D printing within the network-based Industry 4.0 paradigm requires the use of the IoT for semi-automated or automated real-time data analysis. An effective solution to the problem is hampered by the fact that due to the complexity of the system, and there may be several potentially optimal solutions. The use of CI (Computational intelligence) methods opens up the possibility of reconciling individual approaches to 3D printing while partially standardizing the procedure. The use of the ANN model as a support system enables the generation of an optimal parameter layout while increasing the efficiency of the 3D printing planning process as part of the 3D pre-press procedure. The huge number of data sets and the expected high efficiency of their analysis despite the generation of a complex, controlled, multi-sensory information flow across the assembly line (in space), process (in time), and hierarchy (in the organizational structure) require new, more advanced computational models of the 3D printing process. New, more advanced features may also be required due to a new global approach focused on the environment, waste management, and a smaller carbon footprint. The number of key factors will grow, and despite the comprehensive approach to 3D printing technology, managing them all simultaneously will be beyond the capabilities of an engineer or even a group of engineers. Reasonable, user-friendly software should support this.

The most important hypothesis is that the sustainability of 3D printing processes can be supported by CI- and AI (Artificial intelligence)-based solutions. The authors have experience using AI methods to solve technical problems (e.g., in developing classifier models [62] and predictive models [63], in materials selection in ecodesign [64] and material compatibility [65], and in the form of decision trees and neural networks).

Artificial neural networks, which are an approximate simulation of the brain’s information processing ability, are gaining interest as modern and highly advanced computational techniques. ANNs are a machine learning method for mapping and predicting complex relationships between inputs and outputs. They reflect nonlinear relationships between input data that cannot be recognized by conventional methods. There are several types of ANNs, such as feed-forward networks, back-propagation neural networks (BP-NNs), radial function networks, and probabilistic neural networks.

In terms of a theoretical basis, the ANN first consists of an input layer, a hidden layer, and an output layer of neurons with connections between neurons in subsequent layers that are enhanced by similarities in measured inputs. Complex, large, incomplete, noisy datasets can be easily analyzed for different groups based on similarities of measured parameters. The ANN begins its operation by presenting the pattern of the process variable and continues through the activation level to propagate through the hidden layers. The processing unit aggregates the input data and uses the hidden layer transfer function to calculate the response. The generated result is an estimate, but due to better optimization to the function (due to the identified learning process) it is even more accurate than other analogous statistical procedures. Moreover, such multivariate programs greatly increase the sensitivity and specificity of the evaluation/prediction.

In terms of practical applications, this interest is also manifested in the study of materials and technologies used in 3D printing, hence, the use of neural networks as effective tools for solving various problems, especially those characterized by multidimensional and nonlinear dependencies. Thanks to AI, new optimization decision rules are emerging. The huge explosion of data, materials, technologies, and their features in the practical application of the IoT and Industry 4.0 paradigms requires effective analysis methods to deal with inference and prediction of hidden cause–effect relationships between a large set of properties and single or multiple responses, including those based on digital twins. It is possible to find an optimal and innovative way to solve these problems. Practice will show how to develop appropriate predictive models for changing passive and active prevention.

To achieve the goal of our work, we used our own experience in modeling 3D printing processes using artificial neural networks. Despite the considerable emphasis on objectification of 3D printing processes, modeling them with ANNs is rare. So far, ANNs have been popular in the areas of data analysis, prediction, control, clustering, classification and optimization in physics, mechanics, geology, medicine, economics, etc. They usually provide approximate (estimated) results, but allow mapping of complex nonlinear functions, control of multivariate problems including a large number of independent variables, and limited theoretical knowledge is needed for model building.

Many 3D printing parameters are optimized to improve the quality of manufactured objects and their features, but the use of an ANN for this purpose is still limited. Even less common is the use of ANNs to optimize the environmental characteristics of 3D printing. This paper aims to make up for the abovementioned shortcomings. Based on previous publications and our own data sets, two ANNs were constructed: ANN1 to assess electricity consumption, and ANN2 to assess air pollution (risk assessment).

Due to the lack of a model or mathematical representation in both of the above cases, we used a feed-forward neural network with a back-propagation algorithm, the full formulation of which is unknown, to solve these types of problems. We took into account that logistic models, etc., often predict more accurately than neural network models in terms of mean squared error, but such ANN models are better suited to the loss functions associated with the desire to more accurately predict certain combinations of categorical responses than others.

We calculated the mean square error (MSE) as the mean squared difference between estimated and actual values Equation (1):

(1) $$MSE=\frac{1}{n}\sum_{i=1}^{n}{\left(y_{i}-{\hat{y}}_{i}\right)}^{2}$$

where *n* is number of data points, *y_i_*—observed values, and *ŷ_i_*—predicted values.

A multilayer perceptron (MLP) is a class of feed-forward ANNs. We checked both the sigmoidal activation function Equation (2):

(2) $$y(x)={\left(1+e^{-x}\right)}^{-1}$$

and the hyperbolic tangent Equation (3):

(3) y(x)=tanh(x).

**Figure 1:**
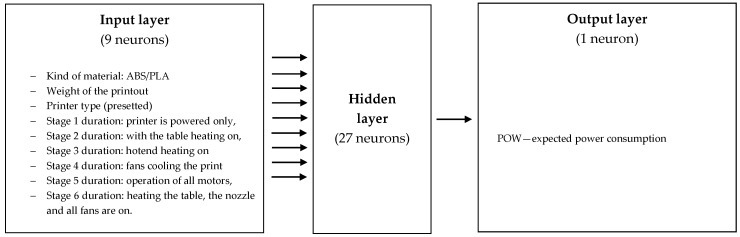
Artificial neural network 1 (ANN1) optimal structure for evaluating energy consumption: inputs and outputs.

**Figure 2:**
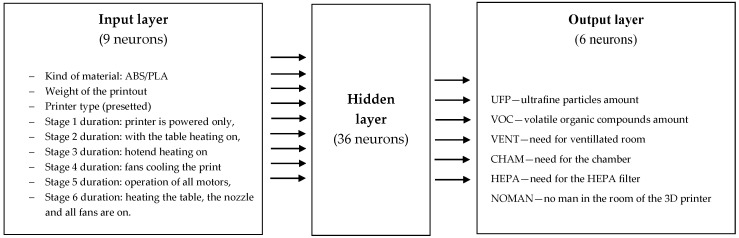
Artificial neural network 2 (ANN2) optimal structure for air pollution assessment structure: inputs and outputs.

MATLAB 16.0 (MathWorks) software was used for training and optimization purposes, including the Statistics and Machine Learning Toolbox, and the Deep Learning Toolbox. The explanatory variables (process characteristics) used in the predictive models are shown in Figure 1 (for ANN1) and Figure 2 (for ANN2). We used data sets from industrial and research practice with 3D printers, in particular, those described in the Introduction of energy consumption during the 3D printing work cycle and air pollution measurements. The input variables were rescaled using the same maximum and minimum values from the sample data. The initial values of the network weights were estimates ranging from –1 to 1. To prevent bias in the weights at start-up, weights randomly selected at initialization were normalized. Two different stopping points were included in the learning process: after 1000 iterations and after 2000 iterations. The samples were divided into three groups: 70% (learning), 20% (testing), and 10% (validation).

## 3. Results

The models selected in the selection process were simpler to construct than they could have been. All results were achieved after 1000 iterations. The best results for ANN1 were achieved for n = 9, m = 1, and 27 neurons in the hidden layer (i.e., MLP 9-27-1), but we found that other ANN1 structures were also effective, such as MLP 9-18-1, MLP 9-36-1, and MLP 9-45-1 (Table 1).

ANN1 was able to minimize the MSE for the data in the training set to very small values (0.001–0.01) (Table 2 and Table 3).

**Figure 3:**
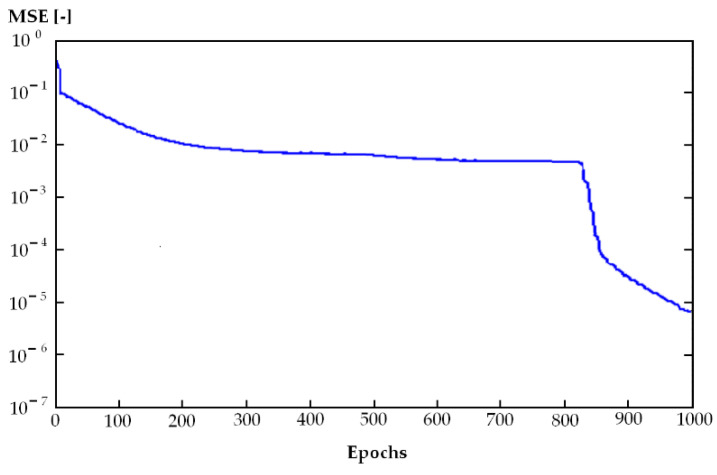
Values of mean square error (MSE) during learning (ANN1), x axis – number of epochs.

The number of learning epochs ranged from 500 to 1000 (Figure 3).

Linking the results of the ANN1 model to 3D printing technology allowed easier evaluation and prediction of energy consumption for different types of 3D printers and related entire 3D printing processes within Industry 4.0. Its short computation time, very good quality (0.9554), and very low MSE (0.001) allowed optimization of the real-world 3D printing process toward greater environmental friendliness.

The best results for ANN2 were achieved for n = 9, m = 6, and 35 neurons in the hidden layer (i.e., MLP 9-35-6), but we found that other ANN2 structures were also effective, such as MLP 9-18-6, MLP 9-27-6, and MLP 9-45-6 (Table 4). Agreeing with our expectations, a higher number of neurons in the hidden layer was necessary to achieve results similar to ANN1 due to the more complicated connectivity to the output layer (6 neurons instead of 1).

**Figure 4:**
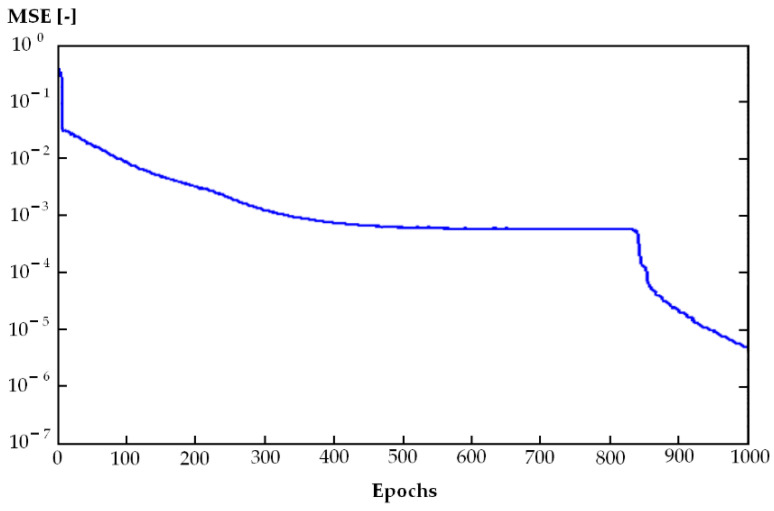
Values of mean square error (MSE) during learning (ANN2), x axis – number of epochs.

The number of learning epochs ranged from 500 to 1000 (Figure 4).

Linking the results of the ANN2 model to 3D printing technology allowed for easier assessment and prediction of air pollution (risk assessment) for different types of 3D printers and associated entire 3D printing processes within Industry 4.0. Its short computational time, very good quality (0.9445), and very low MSE (0.001) allowed for optimization of the real-world 3D printing process toward greater environmental friendliness.

Based on all the ANN models developed, the best network performance was determined: one for each of the individual solutions. In both cases, the best learning quality and best testing efficiency were obtained by the same network, MLP 9-27-1 and MLP 9-35-6, respectively, which also obtained the lowest (R)MSE values.

There are still some limitations that need to be explored, but they need to be tested in practical application(s). The self-learning program will improve the performance of the ANN due to more data input. The proposed solutions do not replace but complement the existing 3D printing meters and software.

## 4. Discussion

The main objective of this paper was to build two proprietary ANN models. The work tested whether an advisory program based on artificial intelligence methods could propose an optimal distribution of 3D printing parameters from the point of view of energy consumption and air pollution, while standardizing the 3D printing planning process. Models developed in this way can be a tool to support the work of engineers in the selection of parameters necessary to plan the 3D printing process. The advantage of the above solution is the possibility to use the knowledge and experience gained so far concerning a diverse group of 3D printing procedures and technologies.

The novelty of the solution stems from the fact that, despite a number of advantages, ANN-based computer-based advisory systems are not routinely used in everyday practice in 3D printing systems. To date, a limited number of publications have investigated the use of ANNs in 3D printing parameter selection and optimization. Research on the sustainable characteristics of materials [66], including polymers used as 3D printing material [67], is ongoing, but the number of studies is so far small. We view our results as preliminary. They lead to further implications: in the broadest context, CI-based optimization of 3D printing processes within the Industry 4.0 paradigm is possible [68]. This requires not only efficient technical solutions, but also correct consumer behavior and attitudes toward the mentioned waste management [69]. Global conclusions regarding the sustainability of 3D printing vary with time and research [70]. A simple calculation of the energy consumption of a 3D printer was proposed by Annibaldi and Rotilio [70], but this research represents another breakthrough in the aforementioned area. Limitations potentially minimizing the use of 3D printing for environmental reasons include high production costs and high energy consumption during the process [71,72,73,74]. The continuous development of 3D printing means that the various printers, technologies, materials, etc., used to evaluate 3D printing technologies may lead to different conclusions within the environmental impact assessment [75]. In our view, this may support our point that CI-based assessment and control can significantly improve safety and reduce the environmental impact of 3D printing. Undoubtedly, there is a need for a more objective environmental profile of 3D printing based on novel assessment models capable of quantitatively reflecting the actual and future environmental burden caused by emerging technologies [76].

Our results confirm the usefulness of CI-based solutions from the perspective of sustainability in 3D printing. They can be interpreted as one of the first steps toward broader automation of additive manufacturing processes. The impact of CI-based approaches may be greater than we previously assumed due to the unique paradigms of Industry 4.0, including the need for technical oversight of products throughout the manufacturing process and rapid response to any failure or unexpected sensor signal. The advanced sensor infrastructure will require not only distributed signal processing, but also flexible real-time response. The required IT infrastructure must be based on CI solutions that can be useful to optimize the whole process [76].

References to global achievements in the field under review highlight the main benefits of 3D printing in terms of sustainability:

- improved resource efficiency achieved by “just in time” products near the point of consumption,
- capacity to produce less waste,
- possibility of printing parts in a shorter period of time,
- extended life of the product thanks to the possibility of the creation of spare parts “on demand”,
- shorter and more local supply chains,
- a reduced carbon footprint of product manufacturing [77].

Potential industrial applications of the proposed software range from early warning systems for potentially harmful air pollution to systems that optimize material consumption, environmental pollution, and energy consumption of entire production lines.

Future research directions in CI-based solutions to sustainability in 3D printing should cover:

- optimization and automation of the whole process [78], e.g., by including an automatic function in the 3D printer software,
- simulation of the whole product life cycle, including filament recycling, and short life cycle applications,
- development of a distributed recycling platform for 3D printing, e.g., that proposed by Chong et al. to achieve the goal of zero waste production [79].

## 5. Conclusions

The 3D printing market is growing at about 25% per year, so the sustainability of 3D printing materials is of great importance for the future. There is a need for further research on selected aspects of 3D printing sustainability, including more complex solutions that take into account entire manufacturing processes within the Industry 4.0 paradigm.

The proposed effective ANNs with simple structures (MLP-9-27-1 and MLP 9-35-6) can contribute to the understanding of the release mechanisms of chemical contaminants from materials used in 3D printers. This is essential to develop effective strategies for exposure assessment and control, prevention of health hazards and risks associated with 3D printing.

The proposed CI-based software is powerful, and it does not replace but rather complements existing 3D printing metrics and software. Its short computation time, very good quality, and very low (R)MSE (0.001 for both MLP-9-27-1 and MLP 9-35-6) allow the real-world 3D printing process to be optimized toward greater environmental friendliness reflected in lower air pollution and energy consumption.

Further CI-based optimization solutions should be more complex, involving more steps in the 3D printing processes—this can more significantly (even up to more than 10%) reduce air pollution, energy, and material consumption during additive manufacturing processes.

## Figures and Tables

**Table 1:** The best MLP (MultiLayer Perceptron) network models for diagnostic measures (bolded is the best, see also Table 2 and Table 3 for more results).

NS	AH	AO
9-18-1	Sigmoid	Sigmoid
**9-27-1**	**Sigmoid**	**Sigmoid**
9-36-1	Sigmoid	Sigmoid
9-45-1	Tanh	Sigmoid

NS—ANN1 Structure, AH—Activation function in the hidden layer, AO—Activation function in the output layer.

**Table 2:** Selected ANN1 quality assessment (bolded is the best).

Network Name	Quality (Learning)	Quality (Testing)
MLP 9-18-1	0.9011	0.9232
**MLP 9-27-1**	**0.9395**	**0.9554**
MLP9-36-1	0.9230	0.9327
MLP 9-45-1	0.9194	0.9332

**Table 3:** (R - root) MSE (Mean squared error) values for three-MLP (MultiLayer Perceptron) neural networks (bolded is the best).

Network Name	MSE
MLP 9-18-1	0.01
**MLP 9-27-1**	**0.001**
MLP 9-36-1	0.02
MLP 9-45-1	0.02

**Table 4:** The best MLP network models for diagnostic measures (bolded is the best, see also Table 5 and Table 6 for more results).

NS	AH	AO
9-18-6	Sigmoid	Sigmoid
9-27-6	Sigmoid	Sigmoid
**9-35-6**	**Sigmoid**	**Sigmoid**
9-45-6	Sigmoid	Sigmoid

NS—ANN2 Structure, AH—Activation function in the hidden layer, AO—Activation function in the output layer.

**Table 5:** Selected ANN2 quality assessment (bolded is the best).

Network Name	Quality (Learning)	Quality (Testing)
MLP 9-18-6	0.8813	0.9091
MLP 9-27-6	0.9112	0.9275
**MLP 9-35-6**	**0.9231**	**0.9445**
MLP 9-45-6	0.9111	0.9223

**Table 6:** (R)MSE values for three-MLP neural networks (bolded is the best).

Network Name	MSE
MLP 9-18-6	0.01
MLP 9-27-6	0.01
**MLP 9-35-6**	**0.001**
MLP 9-45-6	0.01

## Data Availability

Data sharing is not applicable to this article.
